# Heterogeneity of the Pancreatic Beta Cell

**DOI:** 10.3389/fgene.2017.00022

**Published:** 2017-03-06

**Authors:** Giselle Dominguez Gutierrez, Jesper Gromada, Lori Sussel

**Affiliations:** ^1^Regeneron Pharmaceuticals, TarrytownNY, USA; ^2^Barbara Davis Center for Diabetes, University of Colorado, DenverCO, USA

**Keywords:** pancreatic islet, beta cells, heterogeneity

## Abstract

The pancreatic beta cell functions as a key regulator of blood glucose levels by integrating a variety of signals in response to changing metabolic demands. Variations in beta cell identity that translate into functionally different subpopulations represent an interesting mechanism to allow beta cells to efficiently respond to diverse physiological and pathophysiological conditions. Recently, there is emerging evidence that morphological and functional differences between beta cells exist. Furthermore, the ability of novel single cell technologies to characterize the molecular identity of individual beta cells has created a new era in the beta cell field. These studies are providing important novel information about the origin of beta cell heterogeneity, the type and proportions of the different beta cell subpopulations, as well as their intrinsic properties. Furthermore, characterization of different beta cell subpopulations that could variably offer protection from or drive progression of diabetes has important clinical implications in diabetes prevention, beta cell regeneration and stem cell treatments. In this review, we will assess the evidence that supports the existence of heterogeneous populations of beta cells and the factors that could influence their formation. We will also address novel studies using islet single cell analysis that have provided important information toward understanding beta cell heterogeneity and discuss the caveats that may be associated with these new technologies.

## Introduction

The pancreatic beta cell is an essential endocrine cell type whose identity has been traditionally defined by its function: producing, storing and secreting insulin. However, this definition over-simplifies a more complex identity that is finely tuned to efficiently regulate blood glucose levels, while maintaining the ability to adapt to a wide range of stimuli and physiological challenges. Recent studies have spurred an intense debate about whether beta cells represent a single homogeneous population or consist of subpopulations with functional and molecular variations to facilitate specialized tasks and responses to changes in the physiological environment. If beta cell heterogeneity exists, it will be important to determine whether it is a means by which beta cells functionally adapt during normal and pathological conditions and, in particular to the development and progression of diabetes. This could have profound implications for our ability to understand and treat the disease.

In this review, we will discuss the evidence supporting the existence of beta cell heterogeneity, the mechanisms influencing its development, and its functional relevance. We will discuss recent studies that shed light on the molecular aspects of beta cell heterogeneity and exciting areas of research that have yet to be applied to the beta cell field.

## Examples of Cell Heterogeneity

To understand heterogeneity, we need to first consider where it originates, and the advantage of its existence. It is well established that most organisms constantly evolve to successfully adapt to their environment; leaving only the fittest populations with the ability to survive (Darwin, 1859). In order to adapt, cell populations have to find the best strategy with regard to the division of essential functions. Theoretical research on the concept of division of labor within biological systems suggests that populations can be composed of either generalists or specialists; generalists perform all tasks and specialists perform particular ones. Interestingly, it is the populations that balance and divide their tasks into specialized subgroups that seem to be the fittest (Wahl, 2002).

Evidence to support the concept of specialization is present at all levels of nature, with populations dividing into functionally different groups in order to have a survival advantage, to respond timely and efficiently to challenges in the environment, and to ultimately accomplish the purpose of the population as a whole. For example, heterogeneity in the division of labor is exemplified in the partitioning of incompatible yet complementing functions of cyanobacteria (Flores and Herrero, 2010). Adaptation to environmental challenges can also be observed in the ability of bacterial populations to acquire resistance to antibiotics, in the capability of yeast populations to adapt to highly toxic environments, and in the differential rate of infection in cells after viral exposure (Balaban et al., 2004; Bishop et al., 2007; Snijder et al., 2009). Effective and prompt response to challenges can also be observed in the development of subgroups of immune or neural cells destined to respond to insults such as injury or hypoxia (Miklos and Kovacs, 2003; Llorens-Bobadilla et al., 2015; Silvestre-Roig et al., 2016). Heterogeneity has therefore evolved to provide cells with the most efficient way to perform its functions. For this reason, it is essential to consider the ultimate duties a cell has to perform, how these functions are most efficiently carried out and what information the architectural composition and molecular signatures can provide.

## Beta Cell Heterogeneity

Beta cell heterogeneity has recently received increasing attention, and studies are emerging that challenge our understanding of beta cell identity. As we learn more about the potential for variation in the beta cell population, it is important to consider the developmental origin of beta cells, their highly interactive nature, and the variety of input signals they receive. These, among other factors can lead to the development of different types of heterogeneity.

Cell to cell variations exist in several different dimensions ranging from observable physical features to biochemical and molecular fluctuations. While these differences are becoming easier to identify, it is important to determine whether biologically relevant functions are associated with the observed changes in identity. In this regard, the amount of evidence that supports the existence of significant and relevant variation in beta cells has grown stronger in the last decade. Furthermore, several studies have provided clues as to the origin of cellular variations, which are possibly established from the beginning of pancreas and/or endocrine cell differentiation.

## Beta Cell Heterogeneity During Development

In rodents and humans, regional differences in the pancreas exist during the earliest stages of fetal development, with the pancreas arising from two epithelial buds that form from non-contiguous regions of the distal foregut endoderm in response to distinct signaling events (Jennings et al., 2015). As pancreas development progresses, the pancreatic buds fuse to form one organ; however, the ratio of pancreatic endocrine populations display clear regional differences depending on their development origin: the dorsal pancreas gives rise to islets with a higher proportion of somatostatin and glucagon expressing cells, while the ventral pancreas contains islets rich in pancreatic polypeptide cells (Trimble and Renold, 1981; Trimble et al., 1982; Brereton et al., 2015; Jansson et al., 2016). It remains to be determined whether the regional origin of the beta cells from the dorsal versus ventral pancreas also translates into differences of identity in the adult beta cell population. In addition, during human pancreagenesis, at least two distinct populations of endocrine cells are evident: freely dispersed cells and cells that group together to form the islets of Langerhans. To date, the differences or purpose of the freely localized beta cells remains unknown, likely because these cells are lost during standard islet isolation procedures. Nevertheless, both populations contain individual cells that express multiple combinations of the endocrine hormones (polyhormonal) in the early stages; but as development progresses, the beta cells acquire their insulin-expressing monohormonal identity and become functionally mature. As the polyhormonal cells resolve into single insulin-producing cells, it is unknown whether all or subpopulations of beta cells preferentially arise from a particular polyhormonal population. It remains possible that beta cells originate from different polyhormonal combinations, which could result in molecularly and/or functionally distinct beta cell subpopulations. Interestingly, heterogeneity also exists during the maturation event with beta cells located within the core of the islet maturing first, followed by the maturation of cells within the outer part of the islet (Bocian-Sobkowska et al., 1999).

Although fewer polyhormonal cells are present in the developing rodent pancreas, genetic disruption of transcription factor function provides additional evidence for the existence of beta cell heterogeneity during gestation. For example, depletion of Nkx6.1 in endocrine precursor cells results in decreased numbers of insulin+ cells (∼16% compared to 55% in the control mice) and an allocation of the endocrine precursor population into other endocrine identities (Schaffer et al., 2013). Similarly, global deletion of Mafb or Mnx1 renders a partial reduction in beta cell numbers; 50% in the case of Mafb and approximately 65% in the case of Mnx1 (Harrison et al., 1999; Li et al., 1999; Artner et al., 2007). If we assume that all beta cells have a homogeneous origin that is specified by the same transcriptional pathways, we would expect that disruption of these regulatory pathways would result in ablation of the entire beta cell population. Instead, we observe differential beta cell responses to alterations in certain transcriptional pathways that could indicate the formation of subpopulations of beta cells from the earliest developmental stages.

## Morphological Heterogeneity

Indications of heterogeneity in adult beta cells were initially based on morphological features, which included noticeable differences in nuclear size (Hellerstrom et al., 1960). Subsequently, with the application of improved electron microscopy techniques, differences in organelle size and secretory granule content were documented (Stefan et al., 1987; Kiekens et al., 1992; Ling et al., 2006; Katsuta et al., 2012). Furthermore, it appeared that glucose stimulation resulted in functional changes that were initiated in the islet core: Beta cells within the core of the islet de-granulated rapidly and presented with enlarged rough endoplasmic reticulum and Golgi apparatus, while peripheral beta cells only began to de-granulate after sustained glucose stimulation (Stefan et al., 1987). Furthermore, differences in insulin granularity did not only pertain to their location within the islet but also to the identity of their neighboring cells, as beta cells coupled to delta cells showed increased insulin granule content (Pipeleers, 1987).

Evidence of morphological heterogeneity extended beyond the internal features of the beta cell, and into the molecular mechanisms responsible for interacting with other islet cells. Gap junctions, which are important for beta cell coupling, were also found to have heterogeneous expression patterns. Meda et al. (1980) observed twice as many gap junctions in the periphery of the islet under basal conditions; nucleotide transfer and dye coupling experiments later confirmed the heterogeneous nature of coupling mechanisms between beta cells (Meda et al., 1981, 1983). Collectively, differences in morphological features have provided strong evidence for the existence of beta cell heterogeneity at the molecular and functional level.

## Functional Heterogeneity

The existence of functional variations within the beta cell population would offer advantages for their ability to respond robustly to different physiological conditions, especially given the consequences of having insufficient or excess insulin in the body. It is likely that precise regulation of insulin secretion would be difficult to accomplish if all beta cells were homogenous in their glucose sensing and secretion capabilities as they would all respond simultaneously with similar force to a given stimulus, without an inherent ability to quickly adapt to changing conditions. Alternatively, the existence of subpopulations of beta cells with different thresholds for glucose would modulate the response, and insulin secretion would be more tightly regulated.

Differences in the thresholds at which beta cells elicit an insulin secretory response to glucose have been characterized by several investigators. Giordano et al. (1991) observed that beta cells have distinct insulin secretory patterns; with cells clustering into two populations: responsive and unresponsive. Responsive beta cells that secreted insulin during the first glucose challenge also secreted insulin during subsequent stimulation assays. Similarly, unresponsive beta cells remained non-responsive in subsequent assays. Interestingly, in both responding and non-responding populations, approximately 75% of the beta cells displayed fixed responses; while 25% of beta cells fluctuated between an unresponsive and responsive state (Giordano et al., 1991). Several additional studies have also confirmed the existence of beta cell populations that differed in their sensitivity or capacity to respond to glucose (Soria et al., 1991; Kiekens et al., 1992; Van Schravendijk et al., 1992; Ling et al., 1998).

Differential expression or activities of the factors and pathways contributing to the insulin secretory response have also been detected. Jetton and Magnuson (1992) found variation between beta cells in the overall expression levels of glucokinase, a rate-limiting enzyme that regulates one of earliest steps in the glucose response. Furthermore, it has been demonstrated that highly glucose responsive beta cells had a ∼60% increase in glucokinase activity (Heimberg et al., 1993); suggesting that glucose phosphorylation may be a determining factor in influencing beta cell heterogeneity. Pioneering studies by Dean and Matthews (1968) also showed that cells located near the surface of the islet had larger membrane potentials and there is early evidence showing that some but not all beta cells are electrically coupled or synchronized (Meda et al., 1984, 1991). However, assessment of specific electrophysiological characteristics has not identified the relevant effectors of these differences; the individual electrical membranes appear similar despite their ability or inability to respond to glucose (Soria et al., 1991). These data suggest that downstream effectors in the glucose-stimulated insulin secretory process may be important for the development of functional variations. One candidate pathway could be related to the beta cells’ calcium responses, which precede the last steps before insulin granule exocytosis, and have been shown to vary between beta cells (Herchuelz et al., 1991; Jonkers and Henquin, 2001; Benninger and Piston, 2014). These functional studies demonstrate that there is heterogeneity at several levels of the glucose response process; however, it is not known how these differences are ultimately organized and or associated with specific beta cell subpopulations.

A new model describing functional integration of beta cell heterogeneity was recently described Johnston et al. (2016). Through a novel set of experiments, insulin secretion responses were found to be orchestrated by two populations of cells: hub cells that function as pacemakers to dictate the insulin secretion dynamics and follower cells that respond to hub cell signaling cues. Interestingly, hub cells had increased glucokinase levels and the influence of glucokinase was greatly decreased after gap junction inhibition, consistent with previous studies showing differential expression of both glucokinase and gap junction coupling in islet beta cells (Meda et al., 1980, 1981, 1983; Jetton and Magnuson, 1992; Heimberg et al., 1993; Benninger and Piston, 2014). Interestingly, hub cells were more sensitive to diabetic-like insults; likely due to their high metabolic rate and immature phenotype. The driving forces behind these populations remain to be analyzed. Furthermore, it will be important to determine if other functional beta cell groups exist and how they interact with each other and contribute to development and progression of disease.

Equally important to having mechanisms that allow a graded response to different glucose stimuli, would be the ability to impart subpopulations with specialized functions, such as proliferative capacity or responsiveness to non-glucose stimuli, such that the beta cell population would more efficiently respond to the many environmental challenges it routinely encounters. Several lines of evidence have recently emerged to suggest the existence beta cells with specialized capacities. Soria et al. (1991) demonstrated that beta cells that were otherwise unresponsive to glucose could in fact secrete insulin when presented with an appropriate stimulus, such as tolbutamide. Similarly, glucose non-responsive beta cells were shown to exert an insulin secretion response when stimulated with glucagon (Giordano et al., 1991) or glucagon-like peptide 1 (Holz et al., 1993; Gromada et al., 1995). More recently, Flattop (Fltp) was identified as a molecular marker whose expression differentiates populations of beta cells based on their mature or proliferative capacity (Bader et al., 2016). Collectively, these findings suggest there are different layers of functional complexity that exist within the beta cell population that could endow beta cells with differential capabilities to respond to a diverse set of stimuli and challenges.

## Molecular Heterogeneity

**FIGURE 1:**
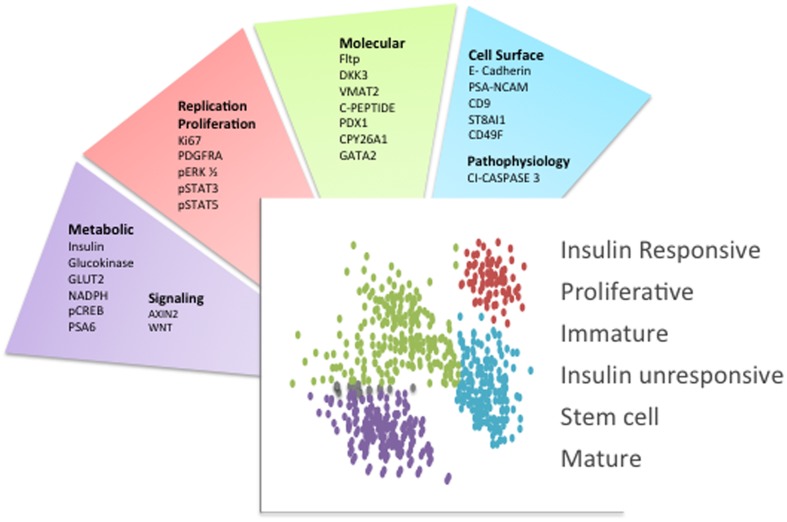
**Beta cell heterogeneity markers.** Schematic indicating the primary molecules that were used to separate islet beta cell subpopulations. Each factor is categorized based on its surface localization or function. The distinct beta cell subpopulations are schematized as different colored dots and the main phenotypic characteristics or functions associated with these beta cell subpopulations are indicated. This schematic summarizes the concepts and respective references that were discussed in the text.

Presently, novel technological advances are creating a new era in the beta cell field, allowing us to identify the molecular fingerprint of individual cells. To begin to lay the foundation for studying beta cells at the molecular level, two approaches have been undertaken: an unbiased analysis of gene expression through single cell RNA sequencing, and the separation of beta cells into groups that differ in their protein expression. Although there are caveats associated with single cell RNA sequencing approaches (Xin et al., 2016a), several studies have provided important new insights into beta cell biology (Bader et al., 2016; Baron et al., 2016; Dorrell et al., 2016; Li et al., 2016; Muraro et al., 2016; Segerstolpe et al., 2016; Wang et al., 2016; Xin et al., 2016b). The separation of cells based on protein expression has also resulted in the discovery of several molecular markers ranging from cell-surface proteins to intracellular molecules involved in metabolism that are differentially expressed in a beta cell subpopulation or denote a particular metabolic state (**Figure 1**) (Pipeleers, 1987; Jetton and Magnuson, 1992; Kiekens et al., 1992; Giordano et al., 1993; Heimberg et al., 1993; Jorns et al., 1999; Guz et al., 2001; Johnson et al., 2006; Bosco et al., 2007; Hermann et al., 2007; Saisho et al., 2008; Wang et al., 2008, 2016; Karaca et al., 2009; Szabat et al., 2009; Smukler et al., 2011; Katsuta et al., 2012; Bader et al., 2016; Beamish et al., 2016; Dorrell et al., 2016).

**FIGURE 2:**
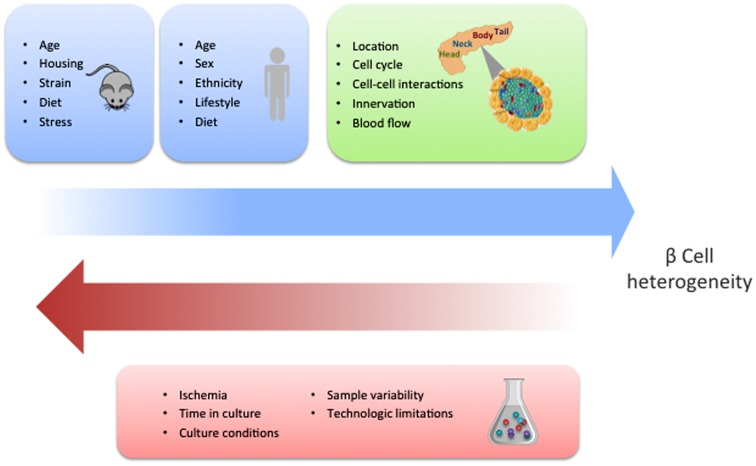
**Potential factors influencing beta cell heterogeneity.** The blue squares indicate the different conditions that have the potential to influence the development of beta cell heterogeneity in mice and human, respectively. This schematic summarizes the concepts that are discussed and referenced in the text. The green square contains the different contextual conditions that may contribute to heterogeneity. Experimental factors with the capacity to dilute or obscure the discovery of beta cell heterogeneity are shown in the red square.

While the identification of specific molecular markers offers a feasible method to characterize distinct beta cell populations, gene expression analyses of single human beta cells have reported contrasting results. Three research groups have described heterogeneity in the beta cell population with particular gene signatures driving such differences (Baron et al., 2016; Muraro et al., 2016; Segerstolpe et al., 2016). However, Xin et al. (2016b) were not able to detect such diverse cell types. Several reasons could account for these discrepancies. In particular, the source and condition of the islets could impact their phenotypes (**Figure 2**). Furthermore, there are clear limitations associated with existing technologies, especially with regards to sensitivity of detection of low abundance transcripts, and the fact that the manipulations required for analysis can easily influence beta cell states. In addition, most of the studies to date have been restricted by the limited access to sufficient numbers of human samples. Ultimately, characterization of larger sample sizes from well controlled populations will allow us to more precisely define the number of discrete beta cell populations based on gene expression and the degree to which the different populations overlap.

Studies of human islets based on the expression of the cell surface antigenic markers CD9 and ST8SA1 have also successfully identified at least four distinct populations of beta cells (Dorrell et al., 2016). However, a study that categorized beta cells based on their expression of factors involved in proliferation, signaling pathways, maturation, and previously identified heterogeneity factors, suggested there were three different beta cell subpopulations (Wang et al., 2016). Again, these differences could be due to the source and/or experimental conditions of the islet cells analyzed (**Figure 2**), in addition to the selection criteria, which may enrich for abundant and rare populations differently. Notably, however, these studies were able to correlate potential functional distinctions between the different beta cell populations, including variations in the expression of *SLC2A2*, an important glucose transporter, and restricted expression of several proliferative factors (PDGFRA, pERK1/2, pSTAT3 and pSTAT5) (Dorrell et al., 2016; Wang et al., 2016). It is interesting that these studies identified many genes without prior association with beta cell biology, several of which appear to be involved in insulin secretion regulation (Dorrell et al., 2016).

Although all of these studies have revealed significant novel information about individual beta cells, it has proven challenging to identify clear cut evidence of the pathways or factors that regulate the molecular aspects behind the variations in beta cell identity and functions. Furthermore, despite the numerous documented gene and protein expression differences, it is intriguing that many of the factors known to regulate diverse beta cell functions are robustly and homogeneously expressed in all beta cells. This would imply that many of the differences between beta cell populations may also involve other layers of molecular complexities, including epigenetic modifications, relative differences in protein levels and/or changes in post-translational modifications. Therefore, it is likely that to fully understand the phenomenon of beta cell heterogeneity at the molecular level, it will be important to characterize the full range of biochemical and molecular differences that make each population unique.

## Context Dependent Factors Influence Heterogeneity

As the existence of beta cell heterogeneity becomes more tangible, it is important to consider what the main drivers of beta cell heterogeneity are. Although heterogeneity could be an intrinsic “context independent” property of the cell based on age or disease states, it is also possible that it could stem from adaption to the environment, including changes in physical interactions and signals received from neighboring cells and tissues (**Figure 2**). Furthermore, alterations in the ratios of different beta cell populations could arise from mechanisms regulating the relative size of the individual populations or interconversion between the different populations. At the organ level, significant differences in the proportion of endocrine cells and pro-hormone processing have been found between the ventral and dorsal pancreas in rodents and humans (Trimble and Renold, 1981; Stefan et al., 1982; Trimble et al., 1982; Rahier et al., 1983; Lee et al., 2010; Wang et al., 2013; Jansson et al., 2016). Proliferation differences have been observed between the duodenal, gastric and splenic region; with the later presenting the most proliferation (Ellenbroek et al., 2013). Differences in insulin degranulation are more evident in splenic regions compared to the duodenum (Stefan et al., 1987); concomitantly, higher rates of insulin biosynthesis and secretion follow the same pattern (Trimble and Renold, 1981; Trimble et al., 1982).

At the islet level, the location of beta cells could also be important for their differential responsiveness. Investigators have shown that not all beta cells are in close contact with neuronal axons (Rodriguez-Diaz et al., 2011), and differences exist in the proximity and access to capillaries, which provide important factors, such as oxygen and nutrients (Bonner-Weir, 1988; Cabrera et al., 2006). Often, such differences depend on islet size and location within the islet (Ballian and Brunicardi, 2007). There are also important influences exerted by the other pancreatic endocrine hormones that affect the beta cell insulin secretion response. Contact between beta and alpha cells increases the insulin secretion output (Pipeleers et al., 1982; Wojtusciszyn et al., 2008); while somatostatin negatively influences insulin secretion (Efendic and Luft, 1975; van der Meulen et al., 2015). Collectively, it is evident that beta cell heterogeneity is likely to exist, with differences that extend from the individual cell level to regional differences at the organ level. Determining the origins and advantages of these variations in beta cell identity, as well as how they are shaped must become a main priority.

## Beta Cell Heterogeneity and Effects on Disease Progression and Therapies

Recognizing that beta cell heterogeneity exists is interesting; however, understanding how beta cell heterogeneity can protect or drive the development of diabetes; as well as how we can apply such information to design disease therapies is the most important goal. To reach this objective we need to understand the consistency and proportion of beta cell subpopulations between different individuals: Do we all possess the same combination of beta cell populations and do the ratios of beta cell populations change with disease or age? Interestingly, the study that identified distinct beta cell subpopulations based on cell surface marker expression determined that all four populations were present in the islets from 17 normal individuals, but the proportions of the subpopulations were shifted in type 2 diabetes patients (Dorrell et al., 2016). However, there was considerable variability between individuals in the ratio of beta cell subpopulations that were identified by single cell mass cytometry. Furthermore, there was no correlation of the different populations between normal and type 2 diabetes donors; however, individual sample populations did show correlation to body mass index, age and diabetes (Wang et al., 2016). The different findings of these two studies are likely not due to the different technologies utilized, but may reflect the quality, variability and small numbers of available human islet samples. This also highlights the importance of additional more highly powered studies using increased numbers of well characterized islet samples before we can make conclusions about beta cell heterogeneity in normal and disease states.

In addition to the concept that changes in beta cell heterogeneity can be caused by age or disease states, it is also worth considering the possibility that certain beta cell populations offer protection or predisposition to diabetes. Accordingly, the difference in the type and proportion of each subpopulation could dictate the likelihood to developing the disease when challenging conditions are encountered. This could help explain why only certain individuals develop diabetes regardless of their metabolic state. Furthermore, it could shed light on the differential responses to treatment that different patients have. It is possible that due to beta cell heterogeneity, individuals will have very different disease etiologies and develop different pathophysiologies. Ultimately, analysis of larger samples sizes that allow for stratification of patient islets, as well as further understanding of the intrinsic properties of each beta cell identity will provide us with answers to these questions that merit significant attention. More importantly, they will give us the necessary tools to develop treatments that are tailored specifically for individual patients.

The existence of beta cell functional heterogeneity could have profound implications on the development of beta cell replacement therapies. Currently, two of the most promising therapies for diabetes include generating alternative sources of beta cells from stem cell populations or stimulating endogenous beta cell proliferation. In the stem cell field, a significant amount of effort is directed toward identifying the appropriate conditions to obtain high quality insulin producing beta–like cells (Pagliuca et al., 2014; Rezania et al., 2014; Yoshihara et al., 2016). However, if different subpopulations of beta cells exist, it will be important to identify which populations being generated *in vitro*, especially if there are subpopulations of beta cells with increased replicative and/or functional capacity. Similarly, identifying endogenous beta cell populations with a higher proliferation propensity could influence the therapies used to stimulate the expansion of beta cells. Ultimately, it will be important to assess the proportions of each subpopulation in different physiological conditions since in order to understand the relative outcomes caused by challenging environmental conditions, disease progression and attempted therapies.

## Future Perspectives

Although the origin and extent of beta cell heterogeneity is still unresolved, novel technologies have allowed us to gain important clues regarding the existence of beta cell heterogeneity and the possible environmental influences. Differences in the proportion of beta cell subpopulations between normal and pathological conditions are still up for debate, and whether these subpopulations are influenced by age or external stimuli, such as diet or body mass index, remains to be determined. However, single cell expression studies have begun to uncover the essential factors and pathways involved in establishing beta cell heterogeneity and will provide the molecular tools to address these questions. In the near future, we should be able to elucidate the connections between molecular, morphological and functional differences within distinct beta cell populations, in addition to determining whether the relative beta cell populations can be altered and by which regulatory mechanisms. Overall, the identification and characterization of beta cell subpopulations, and determination of their relative contribution to function and disease will lead to more effective therapies for the prevention and treatment of diabetes.

## Author Contributions

GG wrote the manuscript based on published data. LS and JG revised the manuscript with GG.

## Conflict of Interest Statement

The authors declare that the research was conducted in the absence of any commercial or financial relationships that could be construed as a potential conflict of interest.
